# Hybrid Scaffolds of Hyaluronic Acid and Collagen Loaded with Prednisolone: an Interesting System for Osteoarthritis

**DOI:** 10.15171/apb.2018.002

**Published:** 2018-03-18

**Authors:** Farhad Mohammadi, Soliman Mohammadi Samani, Nader Tanideh, Fatemeh Ahmadi

**Affiliations:** ^1^Department of Pharmaceutics, School of Pharmacy, Shiraz University of Medical Sciences, Shiraz, Iran.; ^2^Research Center for Nanotechnology in Drug Delivery, School of Pharmacy, Shiraz University of Medical Sciences, Shiraz, Iran.; ^3^Department of Pharmacology, Stem Cell and Transgenic Research Center, Shiraz University of Medical Sciences, Shiraz, Iran.

**Keywords:** Scaffold, Hyaluronic acid, Collagen, Implantable disk, Thermo-sensitive gel, Cartilage regeneration

## Abstract

***Purpose:*** Cartilage regeneration by using polymeric scaffolds is a new option for treatment of osteoarthritis. A good scaffold for tissue engineering should copy the characteristics of natural extracellular matrix. The purpose of this study was to make a dosage form with proper reliability and stability for cartilage repair.

***Methods:*** Hybrid scaffolds containing different ratios of hyaluronic acid (HA) and collagen were prepared and loaded with prednisolone as anti-inflammatory agent. Two different dosage forms (lyophilized implantable disk and thermo-sensitive gels) were examined. A scaffold of cross-linked HA was used as control. Different characterization tests were considered including differential scanning calorimetry (DSC), scanning electron microscopy, mechanical evaluations, and drug release.

***Results:*** The physical and chemical performance of hybrid-scaffolds was better than HA scaffold. Increasing the concentration of HA and collagen improved the physical and chemical characteristics. Regarding the mechanical properties of the hybrid scaffold, the pore size was 20-200µm, compressive modulus was 54.77±0.31 kPa, more than 1200% water uptake was observed after 4 days, gelation temperature was 32±0.16°C, gelation time was 2.4±0.1 min, and drug release was controlled for 5 days by Higuchi release kinetic model.

***Conclusion:*** It seems that this porous hybrid scaffold could be a suitable choice in cartilage regeneration as well as a controlled-release system for delivery of prednisolone in osteoarthritis.

## Introduction

Defects and injuries of the cartilages are significant causes of dysfunction and disability of knee and other organs. Cartilage diseases such as different types of arthritis affect nearly three quarters of the people during the lifetime.^1^ Among the various strategies of treating cartilage defects, tissue engineering has grown rapidly thanks to the application of natural, synthetic or hybrid polymers. They are used in scaffolding as space filling agents, delivery vehicles for proteins or drugs, and 3D structures that establish cells and present the stimuli required for tissue regeneration.^2^

The scaffold material and its characteristics differ according to the type and final application of the tissue.^3^ Some natural materials used as scaffolds are collagen, fibrin, gelatin, agarose, alginate, chitosan and hyaluronic acid (HA). Studies evaluated various synthetic polymers namely poly(acrylic acid) (PAA), poly(propylene fumarate-co-ethylene glycol) (P (PF-co-EG)), poly(ethylene oxide) (PEO), poly(vinyl alcohol) (PVA), poly(lactic-co-glycolic acid) (PLGA), poly(lactic acid) (PLA) and hybrid materials of synthetic and natural polymers.^4,5^

The scaffold structure should be porous which provides the required mechanical stability for adequate cell substitution and facilitates the transfer of oxygen and nutrients. The inflammatory responses and hypersensitivity reactions with scaffolds of natural polymers are not much important due to their biodegradable and biocompatible nature.^6^

Cartilage repair is one of the extensively studied fields in tissue engineering. Simulating the extracellular matrix is a suitable approach for regeneration of cartilage tissue. HA is one of the main constituents of extracellular matrix found in the connective tissue. Collagen is a biocompatible and biodegradable material that helps the adhesion and regeneration of cells.^7^ HA is considered as the main constituent in constructing the preliminary framework. Meanwhile, collagen plays the role of interpenetrated protein fiber in the network to improve the mechanical characteristics of the scaffold and to remain for a longer time in the body and then release the drug in a controlled manner. Lu et al. reported that a combination of these materials was more helpful for cartilage regeneration and provided an appropriate environment for tissue repair.^8^

Corticosteroids reduce the inflammation and pain, especially in moderate to severe cases of osteoarthritis, and improve the joints function for a limited time (about four weeks). Therefore, they can be used as an adjuvant treatment for cartilage repair. To our knowledge there was no report on loading anti-inflammatory drugs in polymeric scaffolds for cartilage tissue regeneration. Thus, the present research used HA and collagen to make a combined polymeric scaffold for cartilage tissue regeneration. The aim was to fabricate a porous and mechanically-stable scaffold loaded with prednisolone as an anti-inflammatory agent. The scaffolds of HA as well as combination of HA and collagen were designed and loaded with drug; and the rheological properties, swelling, degradation and release profiles were studied.

## Materials and Methods

### Preparation of HA and HA/collagen scaffolds

To prepare the HA-based scaffold, 2 ml of distilled water was adjusted to pH 3.5 by dropwise adding of the necessary amounts of acetic acid (Merck; Germany). Two mg prednisolone (Iran Hormone Co., Iran) was added to the solution, and homogenized by using an IKA T10basic, Ultra-Turrax homogenizer (IKA-Werke, Germany) to dissolve. Then, HA (Sigma-Aldrich, Czech Republic) was added to the mixture and the solution was stirred at 1500 rpm for 1 h at 4°C to make an aqueous solution containing 0.1% prednisolone and 1% HA. Poly ethylene glycol diglycidyl ether (Sigma-Aldrich, Czech Republic) as crosslinking agent was added to the aqueous solution to provide a 0.25% solution. The mixture was stirred for 30 min, and then, left to react in a reactor (Labtech, DAIHAN LABTECH) at 35°C for 1 h.

To make disk-shaped HA scaffold, the reacted solution was poured into each well of a 96-well plate (Falcon, USA), frozen at -80°C, and then freeze-dried (CHRIST, Alpha 2-4 LDPlus, Germany). Similar procedure was used to fabricate the combined scaffolds; i.e., 0.1, 0.2 and 0.4% of collagen (Type I, Rat tail, Millipore, Germany) was added to the HA aqueous solution. The obtained mixture was stirred at 1500 rpm for 1 h at 4°C. The same amount of PEGDGE was added to each of the prepared combined aqueous solutions, mixed homogenously, and then permitted to react in the incubator at 35°C for 1 h.

Each reacted mixture was poured in each well of a 96-well plate, frozen at -80°C, and freeze dried. In order to prepare an injectable dosage form, each formulation was added to an aqueous solution containing 18% of poloxamer 407(Sigma-Aldrich; Germany) at 25°C and stirred to provide a thermo-sensitive gel. Finally, the gels were sterilized by filtration through 0.22-µ filter. Table 1 shows the concentration of HA and collagen in different formulations prepared.

**Table 1:** Composition of formulations and results of physical tests

**Formulations**	**HA (mg/ml)**	**Collagen (mg/ml)**	**Compressive modulus (kPa)**	**Gelation temperature (°C)**	**Gelation time (min)**
**F**_1_	10	-	41.26 ± 0.35	33 ± 0.18	3.5 ± 0.07
**F**_2_	5	1	39.86 ± 0.20*	37 ± 0.21*	4.5 ± 0.04*
**F**_3_	10	1	46.37 ± 0.56*	34 ± 0.09*	3.8 ± 0.01*
**F**_4_	10	2	48.43 ± 0.18*	35 ± 0.12*	4 ± 0.08*
**F**_5_	10	4	50.94 ± 0.36*	37 ± 0.33*	5.2 ± 0.11*
**F**_6_	20	1	54.77 ± 0.31*	32 ± 0.16*	2.4 ± 0.1*

Formulations F_2_ to F_6_ showed significant difference in comparison to F_1_. (*p-value<0.05)

### Differential scanning calorimetry

In order to determine whether HA cross-linked and made the network, differential scanning calorimetry (DSC) was done. Thermal analysis was carried out by using DSC 302 (BAHR Thermoanalyse GmbH, Germany). 10 mg HA, 20 µl PEGDGE and 20 mg cross-linked HA were hermetically sealed within the DSC pans and heated in the range of 25-300°C at a rate of 10°C/min under 60 cc/min of nitrogen gas flow; Al_2_O_3_ was used as the reference material. All samples were tested in triplicates.^9^

### Surface morphology of implantable disks

To observe the surface morphology of the prepared disks, scaffolds were scanned by a scanning electron microscope (SEM, S-800, HITACHI, Tokyo, Japan). Prior to scanning, each sample was coated with a thin layer of gold within an ion sputter. Observation was done by 200 x magnification at an alternating voltage of 5 KV. The pore size of the lyophilized disks was measured from SEM images using Digimizer software, version 4.1.1.0 (MedCalc Software).^10^

### Compression test of implantable disks

Cylindrical lyophilized hydrogels (10×3 mm diameter×height) were prepared for compression tests. Lyophilized disks were swelled in distilled water for 2 h and then compressed at constant force rate of 0.1N/minute up to a maximum of 2 N at room temperature by CT3 Texture Analyzer (Brookfield, USA). The test was done three times for each formulation on different samples. The specific software of instrument was used to calculate the compressive modulus from stress-strain curve.^10^

### Swelling measurement of implantable disks

The lyophilized disks were weighed and immersed in distilled water at 25°C. At predetermined times, one of the disks was removed, wiped with ﬁlter paper to remove the excess water, and weighed again. The water uptake of lyophilized hydrogels was calculated by Equation 1:

**Equation 1:** % Water uptake = W_t_-W_0_/W_0_× 100

Herein, W_0_ was the weight of the lyophilized sample and W_t_ was the weight of the swollen sample.^10,11^

### In vitro degradation test of implantable disks

The lyophilized cylindrical hydrogels prepared with different concentrations of HA and collagen, were placed in a 50-ml glass beaker containing 20 ml phosphate buffered saline (PBS). The samples were shaken in a shaker incubator at 50 rpm at 37.5°C. On 1, 4, 7, 14, 28 days after starting the test, each scaffold was removed and rinsed with distilled water, freeze-dried, and then weighed. Percentage of the remaining lyophilized hydrogels was calculated based on Equation 2.

**Equation 2:** % Degradation (t) = Wd_(0)_ –Wd_(t)_/Wd_(0)_ × 100

In this equation, W_d_(0) was the weight of the lyophilized sample and W_d_(t) was the weight of the sample removed at the predetermined time.^12^

### Measurement of gelation temperature for thermo-sensitive gels

A glass beaker containing 50 ml of the thermo-sensitive gel was connected to a water circulator bath. A temperature sensor was connected to a digital thermometer probe and immersed in the gel. The heating rate was 1°C/min and stirring rate was set to 80 rpm. Gelation temperature was defined as the temperature showed on the thermometer when the magnetic bar stopped rotating due to gel formation.^13^

### Measurement of gelation time for thermo-sensitive gels

To evaluate the gelation time of thermo-sensitive gel formulations by the inverted tube test, 900 µl of PBS, equilibrated at 37°C, was poured into a 2-ml microcentrifuge tube (QC LAB, Southampton, UK). 100 µl of the gel solution was poured into the tube and incubated at 37°C. At 2, 5, 10, 15, and 20 minute intervals, the tubes were inverted to verify possible flowability of the gel. The time at which the gel did not flow was considered as the gelation time.^13^

### Drug release measurement

The release tests were performed by the membrane-less model since this method allows direct contact between dosage forms (gels and implantable disks) and release medium; thus, the erosion of dosage forms can be studied.^14^ The dosage forms were placed in 25 ml beakers, and 20 ml PBS solution containing 0.1% sodium lauryl sulfate (SLS) equilibrated at 37°C was poured precisely. The beakers were put in the shaker incubator (Jal Tajhiz, JSH20LUR, Iran) at 37±0.05°C.

The samples were withdrawn at predetermined time points; 0.5, 2, 6, 12, 18 hours and 1, 2, 3, 4, 5 days, and the release medium was completely replaced with fresh medium kept at 37 °C. The concentration of prednisolone in each sample was quantified by high-performance liquid chromatography (HPLC) (CECIL, CE 4200) in 243 nm. The results were shown as percentage of prednisolone released against time. A similar study was performed for prednisolone powder dissolved in release media to prove that the sink condition was provided. All experiments were done in triplicate.

### Statistical analyses

Quantitative results were expressed as mean±SD. The statistical differences were analyzed by ANOVA test, and p-values < 0.05 were considered significant.

## Results and Discussion

Cartilage diseases and defects like osteoarthritis or rheumatoid arthritis are the most important causes of knee and low back pain. Cartilage degenerative diseases usually need surgeries or other invasive procedures. The use of tissue engineering and biomaterials improve the outcome of therapeutic procedures. Use of biomaterials as scaffolds allows the stem cells or growth factors to attach to the scaffold surface or be load into it.^15^ Although previous studies evaluated the effect of prednisolone on treatment of osteoarthritis, this material was never loaded into the combined polymeric formulations. The present study developed a porous and mechanically-stable combined scaffold loaded with prednisolone as an anti-inflammatory agent in two usable dosage forms.

### DSC study

**Figure 1:**
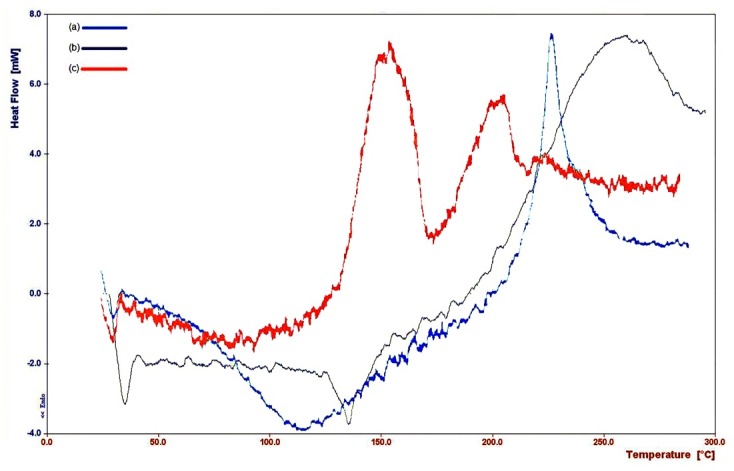
DSC thermograms of (a) HA, (b) PEGDGE, (c) crosslinked HA

Distinct difference was observed between DSC thermograms of HA and cross-linked HA. Figure 1 shows the thermograms of HA, PEGDGE and cross-linked HA. In the HA thermogram, a wide endothermic peak was seen about 100°C which was related to the loss of moisture after the early drying process. About 250°C, a sharp exothermic peak was seen which could possibly be related to the degradation of HA.

In the thermogram of cross-linked HA, sharp exothermic peaks were also observed at higher temperatures due to the increase in chemical stability of the polymer after crosslinking. Seemingly, the first peak revealed conversion into a less-ordered phase, and the second showed thermal degradation. In the thermogram of PEGDGE, an exothermic peak with the onset about 200°C might be associated with flashing of PEGDGE. Similar results were reported by Collins and Birkinshaw.^16,17^ Comparison of the thermograms (a) and (c) in Figure 1 revealed that HA was cross-linked under the reaction condition.

### Surface morphology of implantable disks

**Figure 2:**
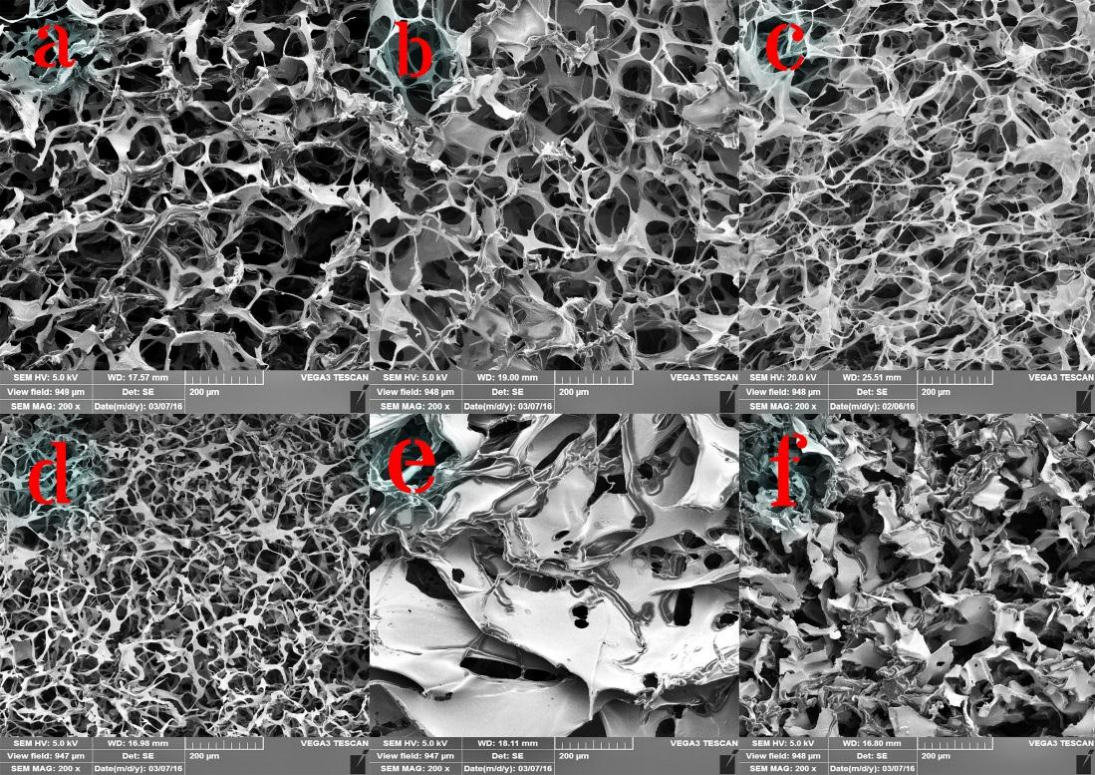
SEM images of HA and HA/collagen scaffolds with different HA and collagen concentrations. (a) F_1_, (b) F_2_, (c) F_3_, (d) F_4_, (e) F_5_, (f) F_6_.

Figure 2 shows the morphology of implantable disks with different concentrations of HA and collagen. SEM images exhibited homogeneous distribution and interconnected porous structure of the implantable disks. Pore size of the implantable disks ranged 20-200 µm. The pore sizes between 100-500 µm can improve the cell proliferation, but the smaller sizes make the implantable disks more compact and more stable.^18^ The existence of pores in scaffolds is one of the perquisites for a scaffold used in tissue engineering, since the pores help passage of necessary nutrients during the growth of tissues and cells.^19^

In the current study, the pore size decreased by increasing the concentration of HA and collagen, because the concentration of HA as main network backbone fibers and collagen as interpenetrated fibers increased simultaneously. The results are parallel with those of the previous studies on HA scaffolds.^20^ Surface area of the porous framework is a significant feature in the proliferation and differentiation of cells. Increasing the concentration of HA and collagen decreased the pore size, which caused increment in the surface area of the implantable disks. However, the strength and stability of implantable disks are important only for a few weeks because a controlled release dosage form is required. Therefore, a balance between these two characteristics should be addressed.

### Compression test of implantable disks

Table 1 presents the compressive modulus of implantable disks calculated by mechanical measurements. The nominal stress (σ_nom_) and strain (ε) were determined by Equation 3.

**Equation 3:** σnom = F/A_0_

**Equation 4:** ε = 1- λ

**Equation 5:** σnom = G (λ-λ^-2^)

**Figure 3:**
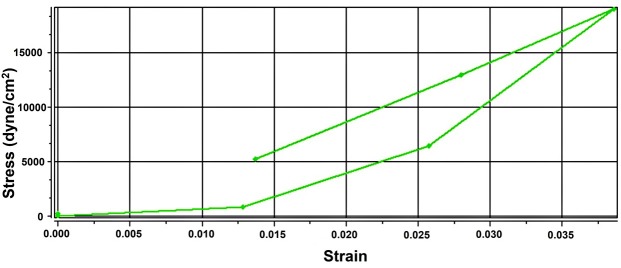
Stress-strain curve of F_6_ formulation.

In these equations, F is the force required to compress the disks, A_0_ is the cross-sectional area of the disks and λ is the deformation ratio (deformed length/initial length). Compressive modulus (G) factor were computed as a ratio of the stress and (λ- λ^-2^) in the linear region of the curves at 20% strain. For example, Figure 3 shows the stress-strain curve of formulation 6. Addition of collagen to the formulation made it stronger than the disk fabricated with HA per se. increasing the concentration of HA and collagen increased the compressive modulus, which consequently provided higher strength against compressive strain. Nonetheless, increasing the HA concentration is more effective on the strength of formulations than increasing the collagen concentration. Zhang et al.,^10^ Kim et al.,^12^ Chen et al.^21^ and Vikingsson et al.^22^ reported similar results.

Cartilage as a tissue that is affected by different stresses like compressive, strain and tension, should be strong enough to resist these stresses. The negative charges of HA and collagen attract water molecules. This hydration effect increased the compressive strength of the tissue. Moreover, collagen is a kind of fibrous protein with negative charge in which three polypeptide chains form a stable helical structure. Thus, increase in the concentrations of HA and collagen improves the mechanical properties.^12^

### Swelling measurement of implantable disks

**Figure 4:**
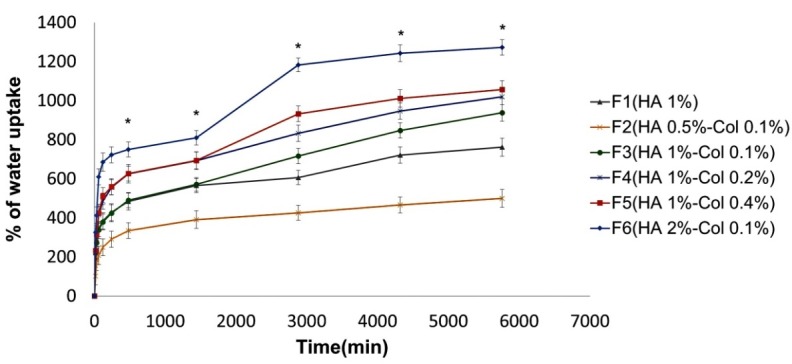
Swelling profile of implantable disk formulations F_1_-F_6_. Formulations F_2_ to F_6_ showed significant difference at determined times compared with F_1_. (*p-value <0.05)

The swelling proﬁles of implantable disks are shown in Figure 4. Accordingly, the percentage of water uptake was directly related to the concentration of HA and collagen. The effect of HA was higher and increasing the HA concentration caused higher percentage of swelling. Both HA and collagen are charged molecules and hydrophilic materials which attract water molecules; so, raising the concentration of HA and collagen increases the amount of water uptake. It seems that the polysaccharide structure of HA totally presents higher number of charges than the three polypeptide chains of collagen. The percentage of swelling affects the diffusion of signaling molecules and nutrients in scaffolds; hence, the higher concentration of HA and collagen would be preferred.^23^

### In vitro degradation test of implantable disks

**Figure 5:**
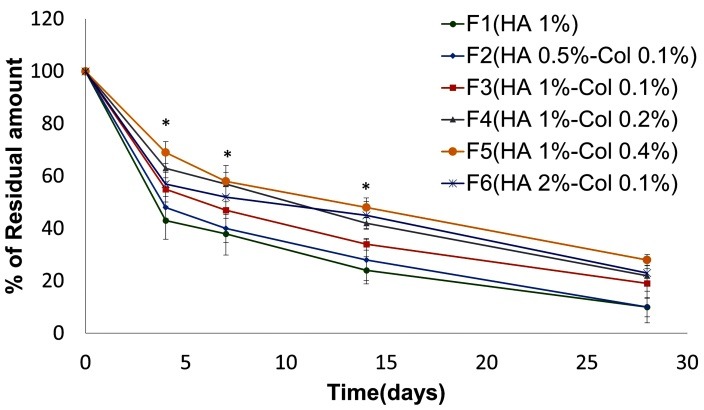
Degradation profile of implantable disk formulations F_1_-F_6_. Formulations F_2_ to F_6_ showed significant difference at determined times in comparison to F_1_. (*p-value <0.05)

Figure 5 shows the degradation of implantable disks of formulations 1 to 6 in PBS at 37°C. Accordingly, increase in the concentration of HA and collagen decreased the amount and rate of degradation. It could be supposed that increase in the concentration of HA and collagen caused the polymer chains to be more ordered and massive, and consequently enhanced the durability of the structure, as proved in SEM images.^24^ The effect of HA and collagen concentration in this test was almost similar; meanwhile, collagen was marginally more effective in stability of implantable disks.

### Gelation temperature of thermo-sensitive gels

Results of measuring the gelation temperature of the gel formulations are presented in Table 1. All formulations would be converted into gel in the body after injection. It was detected that, increasing the HA concentration resulted in reduced gelation temperature; and inversely, increasing the collagen concentration resulted in increased gelation temperature.

### Gelation time of thermo-sensitive gels

Table 1 shows the results of gelation time test. This test was done at 37°C and the results showed that all formulations converted to gel at the body temperature in less than five minutes. By increasing the HA concentration, the gelation time decreased; whereas, increasing the collagen concentration resulted in increased gelation time.

As the temperature increases, dehydration of hydrophobic polyoxypropylene blocks causes poloxamer molecules aggregate and change into micelles; which is the initial step in the gelation process. These spherical micelles have a dehydrated polyoxypropylene core with an outer layer of hydrated swollen polyoxyethylene chains. This micellization occurs when the samples are adequately concentrated. Organized packing of micelles causes gelation. Collagen might be able to interact with micelles, make bonds, and cause delay in the gelation process. Actually, after injection, a short gelation time would be useful in order to decrease the risk of dilution with physiological fluids and local depletion at the injection site.^25^

### Drug release measurement

**Figure 6:**
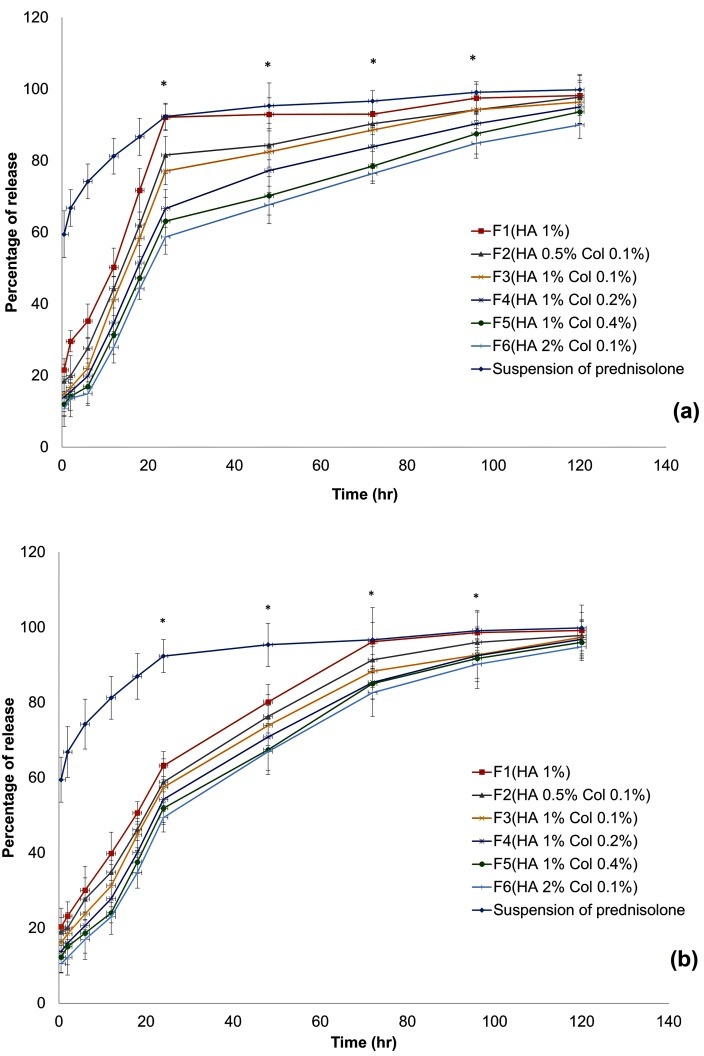
(a) Release profiles of implantable disk formulations F_1_-F_6_. (b) Release profiles of gel formulations F_1_-F_6_. Formulations F_2_ to F_6_ showed significant difference at determined times compared with F_1_. (*p-value <0.05)

Intrinsic solubility of prednisolone in water is 1:1300; but, use of sodium lauryl sulfate (SLS) increased this ratio up to 1:100 and provided the sink condition.^26^ Prednisolone powder was dissolved in the dissolution medium, and the samples were withdrawn and quantified at predetermined time points. It was done to show that the prednisolone release in sustained manner was not related to the limited dissolution rate of prednisolone (Figure 6).

Having separately tested the gels and implantable disks (Figure 6), it was found that at least 60% of the prednisolone was dissolved in the first point of the dissolution profile. It can be concluded that at least within the first 24 h, the prednisolone release from dosage forms was not related to low solubility of prednisolone.^27^

Figure 6 depicts some general trends like the fact that all the formulations were able to sustain the release of drug for more than 100 h. The release of the drug from these formulations was completed within 120 h. Increasing the concentration of HA and collagen resulted in significant decrease of prednisolone release. Although the effect of HA and collagen concentration on the release kinetic was the same, HA concentration provided more pronounced effects. This might be related to the structure of the scaffolds whose main backbone is HA, while prednisolone was just entrapped in this network. Release of drug from gel formulations was slower than implantable disk formulations, because poloxamer creates an additional barrier around the gel formulations and causes slower water penetration and consequently more long-lasting release behavior could be seen.

Korsmeyer-Peppas release model is often used to analyze the release mechanism of polymeric pharmaceutical dosage forms. This model is of great help particularly when the release mechanism is not known or when more than one type of release phenomena is involved. Kinetic of release for all formulations was better fit to Higuchi release kinetic based on the regression coefficients square calculated in Table 2. Based on the release data and fitting experimental results of M_t_ and M_∞_ in the time range, diffusion exponent (n) for the two dosage forms was calculated (Equation 6) to be 0-0.5 (0<n<0.5).

It seems that the release mechanism for all scaffolds were the Fickian diffusion. This mechanism describes the condition in which the rate of diffusion is slower than the rate of chain relaxation.^13^ Based on the degradation and swelling profiles of implantable disks in the first five days, about 50% of each disk was remained and swelled by more than 200% during the studied period. It could be concluded that these hybrid polymeric dosage forms are matrix systems that the drug transport and release through them is mainly provided by diffusion mechanism.^28-30^

**Equation 6:** M_t_/M_∞_ = kt^n^

Herein, n is diffusional exponent, k is kinetic parameter, M_t_ and M_∞_ are the absolute cumulative amounts of prednisolone released at time t and infinite time using the Korsmeyer-Peppas model.^31^

### Statistical Analysis

According to the results of different tests and one-way ANOVA, the formulation F_6_ of implantable disks and thermo-sensitive gels was significantly different from other formulations and was chosen to be examined in the ongoing *in vivo* study.

**Table 2:** Results of fitting release data of implantable disk and gel formulations to different kinetic models.

**Formulations**	**R** ^ 2 ^_0i_	**R** ^ 2 ^_0g_	**R** ^ 2 ^_1i_	**R** ^ 2 ^_1g_	**R** ^ 2 ^_Hi_	**R** ^ 2 ^_Hg_	**R** ^ 2 ^_pi_	**R** ^ 2 ^_pg_	**k**_pi_	**k**_pg_	**n**_pi_	**n**_pg_
**F1**	0.9575	0.9781	0.977	0.9852	0.9831	0.9887	0.897	0.8919	0.24	0.21	0.3519	0.283
**F2**	0.971	0.9713	0.9834	0.9875	0.9841	0.9831	0.8601	0.8568	0.18	0.19	0.3848	0.2808
**F3**	0.9682	0.9656	0.9835	0.9742	0.991	0.9831	0.8447	0.8473	0.15	0.16	0.4271	0.3127
**F4**	0.9553	0.9781	0.9871	0.9857	0.9899	0.9871	0.8324	0.8523	0.14	0.14	0.4011	0.3326
**F5**	0.9792	0.9603	0.9835	0.9715	0.9864	0.9894	0.8265	0.8304	0.12	0.13	0.4172	0.3393
**F6**	0.962	0.9593	0.9805	0.9752	0.9901	0.9922	0.8177	0.8519	0.11	0.11	0.4226	0.3763

R^2^_0i_ = Regression coefficient square of zero order model for implantable disks; R^2^_0g_ = Regression coefficient square of zero order model for gels; R^2^_1i_ = Regression coefficient square of first order model for implantable disks; R^2^_1g_ = Regression coefficient square of first order model for gels; R^2^_Hi_ = Regression coefficient square of Higuchi model for implantable disks; R^2^_Hg_ = Regression coefficient square of Higuchi model for gels; R^2^_pi_ = Regression coefficient square of exponential model for implantable disks; R^2^_pg_ = Regression coefficient square of exponential model for gels; k_pi_ = kinetic parameter of exponential model for implantable disks; k_pg_ = kinetic parameter of exponential model for gels; n_pi_ = diffusion exponent of exponential model for implantable disks; n_pg_ = diffusion exponent of exponential model for gels.

## Conclusion

Based on the findings of this study, it can be concluded that hybrid formulations are superior to formulations containing HA per se. All of the formulated scaffolds were porous and had interconnected pores, suggesting that the scaffolds are useful for the growth of cells and tissues. Furthermore, increasing the concentration of HA and collagen improves the mechanical properties.

Regarding the release test, gel formulations showed more sustained release profile. However, both systems showed Higuchi release kinetic at least for the first 24 h. It can be claimed that hybrid scaffolds (implantable lyophilized disk and injectable gel) can be easily applied as useful dosage forms for cartilage tissue regeneration.

## Acknowledgments

This paper was part of a thesis written by Farhad Mohammadi for the partial fulfillment of PhD degree. It was financially supported by Shiraz University of Medical Sciences (Grant# 94-7585).

## Ethical Issues

Not applicable.

## Conflict of Interest

The authors declare no conflict of interests.
